# Network Pharmacology Analyses of the Pharmacological Targets and Therapeutic Mechanisms of Salvianolic Acid A in Myocardial Infarction

**DOI:** 10.1155/2022/8954035

**Published:** 2022-10-05

**Authors:** Qing Huang, Chao Zhang, Shaoyong Tang, Xiaoyan Wu, Xiong Peng

**Affiliations:** ^1^Department of Cardiology, Wuhan Fourth Hospital, Wuhan, Hubei, China; ^2^Heart Function Testing Center of Cardiovascular Medicine, Zhongnan Hospital of Wuhan University, Wuhan, Hubei, China; ^3^Department of Cardiology, Zhongnan Hospital of Wuhan University, Wuhan, Hubei, China

## Abstract

**Objective:**

Salvianolic acid A, a natural polyphenolic ingredient extracted from traditional Chinese medicine, possesses an excellent pharmacological activity against cardiovascular diseases. Herein, therapeutic mechanisms of salvianolic acid A in myocardial infarction were explored through systematic and comprehensive network pharmacology analyses.

**Methods:**

The chemical structure of salvianolic acid A was retrieved from PubChem database. Targets of salvianolic acid A were estimated through SwissTargetPrediction, HERB, and TargetNet databases. Additionally, by GeneCards, OMIM, DisGeNET, and TTD online tools, myocardial infarction-relevant targets were predicted. Following intersection, therapeutic targets were determined. The interaction of their products was evaluated with STRING database, and hub therapeutic targets were selected. GO and KEGG enrichment analyses of therapeutic targets were then implemented. H9C2 cells were exposed to oxygen‐glucose deprivation/reoxygenation (OGD/R) to mimic myocardial infarction and administrated with salvianolic acid A. Cellular proliferation was assayed via CCK‐8 assay, and hub therapeutic targets were verified with RT-qPCR.

**Results:**

In total, 120 therapeutic targets of salvianolic acid A in myocardial infarction were identified. There were close interactions between their products. Ten hub therapeutic targets were determined, covering SRC, CTNNB1, PIK3CA, AKT1, RELA, EGFR, FYN, ITGB1, MAPK8, and NFKB1. Therapeutic targets were significantly correlated to myocardial infarction-relevant pathways, especially PI3K-Akt signaling pathway. Salvianolic acid A administration remarkably ameliorated the viability of OGD/R-induced H9C2 cells, and altered the expression of hub therapeutic targets.

**Conclusion:**

Our work uncovers therapeutic mechanisms of salvianolic acid A for the treatment of myocardial infarction, providing a new insight into further research on salvianolic acid A.

## 1. Introduction

Cardiovascular disease is a general term for diseases that damage the heart and blood vessels, characterized by rapid onset and high morbidity [1]. In accordance with the WHO, approximately 18 million individuals died from cardiovascular disease in 2019, which represented 32% of overall global deaths as the dominating cause of deaths in all diseases [2]. Myocardial infarction, often triggered by blood clot blocking artery or bypass graft, has the features of a sudden decrease in blood flow to myocardium, eventually resulting in heart failure even death [3]. Among cardiovascular diseases, myocardial infarction has become a primary international health issue [4]. Thrombolysis, percutaneous coronary intervention as well as coronary artery bypass graft remain the most commonly applied approaches in treating myocardial infarction. Nevertheless, patients often present complications such as bleeding, ischemia-reperfusion damage as well as coronary restenosis. Therefore, more effective therapeutic approaches to alleviate apoptosis of cardiomyocytes and facilitate local angiogenesis are urgently required for preventing the expansion of irreversible myocardial damage [5].

Salvianolic acids are extracted from *Salvia miltiorrhiza Bunge* (Danshen). Salvianolic acid A is the strongest antioxidant among salvianolic acids, which is an effective free radical scavenger because of the polyphenolic structure [6]. It has been suggested that salvianolic acid A exerts diverse pharmacological properties, especially for cardiovascular diseases [7]. Preclinical evidence has demonstrated the cardioprotective property of salvianolic acid A against myocardial infarction. Salvianolic acid A attenuates myocardial infarction-triggered apoptosis and inflammation through activation of thioredoxin [8]. Also, it exhibits the antiapoptotic and cardioprotective effects on rat cardiomyocytes under ischemia/reperfusion via DUSP-induced modulation of ERK1/2/JNK signaling [9]. Experimental evidence also demonstrates that salvianolic acid A possesses antioxidant activity and exerts a remarkable protective function against isoproterenol-triggered myocardial infarction [10]. Additionally, salvianolic acid A exhibits cardioprotective effects by facilitating angiogenesis [11] as well as lowering plasma uric acid levels [12] and plasma and tissue dimethylarginine levels [13] in acute myocardial infarction animal models. Despite this, there is a lack of systematic and comprehensive analysis of the therapeutic mechanisms of salvianolic acid A in myocardial infarction.

Network pharmacology is an effective approach to establish a “compound-protein/gene-disease” network, which reveals the regulation principle of small molecule compounds with a high-throughput manner, thus providing a broader selection of pharmacologically relevant targets [14]. Herein, we applied network pharmacology analyses to dissect the therapeutic mechanisms of salvianolic acid A systematically and comprehensively in myocardial infarction. Additionally, the effects and therapeutic targets of salvianolic acid A in myocardial infarction were verified in oxygen‐glucose deprivation/reoxygenation (OGD/R)-induced H9C2 cells that mimicked myocardial infarction.

## 2. Materials and Methods

### 2.1. Retrieval of the Chemical Structure of Salvianolic Acid A

PubChem (https://pubchem.ncbi.nlm.nih.gov) is an important chemical information resource, which comprises over 293 million depositor-provided substance descriptions, 111 million unique chemical structures as well as 271 million biological activity data points from 1.2 million bioassay experiments [15]. The chemical structure of salvianolic acid A was accessed from PubChem.

### 2.2. Analyses of Salvianolic Acid A Targets

The SwissTargetPrediction web tool (http://www.swisstargetprediction.ch) allows users to predict the most possible macromolecular targets of a specific small molecule compound on the basis of 2D and 3D similarity with a library of 370000 known actives on over 3000 proteins from three distinct species [16]. The canonical SMILES “C1=CC(=C(C=C1CC(C(=O)O)OC(=O)C=CC2=C(C(=C(C=C2)O)O)C=CC3=CC(=C(C=C3)O)O)O)O” of salvianolic acid A was uploaded to SwissTargetPrediction, and potential molecular targets of salvianolic acid A were downloaded. HERB (http://herb.ac.cn/) is a high-throughput experiment and reference guide database of traditional Chinese medicine [17]. Salvianolic acid A ingredient was input into HERB database, and relevant gene targets were gathered from curated references. TargetNet (http://targetnet.scbdd.com) is a web service to estimate potential drug-target interactions through multitarget SAR models [18]. Potential targets of salvianolic acid A were screened on the basis of ECFP2 molecular fingerprint in accordance with the area under the receiver operating characteristic curve = 1. On the basis of SwissTargetPrediction, HERB, and TargetNet databases, potential molecular targets of salvianolic acid A were merged and deduplicated. Through the Uniprot database (https://www.uniprot.org/) [19], gene name of targets was corrected and matched.

### 2.3. Acquirement of Targets of Myocardial Infarction

GeneCards (http://www.genecards.org/) [20] and Online Mendelian Inheritance in Man (OMIM; http://omim.org) [21] are comprehensive, and authoritative research resources of annotative information of human genes. DisGeNET (http://www.disgenet.org/) is a knowledge management platform that integrates and standardizes data about disease-relevant genes and variants from a variety of sources, covering over 24,000 diseases and phenotypes, 17,000 genes as well as 117,000 genomic variants [22]. Therapeutic Target Database (TTD; http://db.idrblab.net/ttd/) is a popular information resource of the known therapeutic protein and nucleic acid targets, the targeted diseases, the pathway information as well as the matched drugs or ligands [23]. Potential targets of myocardial infarction were searched from above databases, followed by merging, deduplication, and correction through the Uniprot database.

### 2.4. Identification of Targets Shared by Salvianolic Acid A and Myocardial Infarction

Targets of salvianolic acid A and myocardial infarction were intersected, and imported into Venny 2.1 online tool (http://bioinfogp.cnb.csic.es/tools/venny/index.html). The Venn diagram of targets shared by salvianolic acid A and myocardial infarction was drawn.

### 2.5. Protein-Protein Interaction (PPI) Analyses

The PPIs of myocardial infarction targets of salvianolic acid A were estimated with the STRING online tool (https://string-db.org/) that integrates all known and predicted PPIs comprising physical and functional interactions [24]. The criteria included organism, Homo sapiens; settings, highest confidence (0.900). The PPI network was drawn via Cytoscape 3.7.2 (https://cytoscape.org/) [25]. The degree of targets was computed with Count package. Through cytoHubba plugin, hub genes were selected.

### 2.6. Functional Enrichment Analyses

Utilizing clusterProfiler package [26], functional enrichment analyses of myocardial infarction targets of salvianolic acid A were implemented. Gene ontology (GO) analyses were utilized for describing biological functions of gene products, comprising biological processes (BPs), cellular components (CCs), along with molecular functions (MFs). Kyoto Encyclopedia of Genes and Genomes (KEGG) pathway enrichment analyses were applied for probing out signaling pathways enriched by myocardial infarction targets of salvianolic acid A. A *P* value < 0.05 was regarded as significant enrichment. The map of KEGG pathways was drawn via pathview package [27].

### 2.7. Cell Culture, OGD/R Injury, and Administration

H9C2 cells (ATCC, USA) were maintained in DMEM (Gibco, USA) with 10% fetal bovine serum, penicillin (100 *μ*g/mL), and streptomycin (100 *μ*g/mL) in a humidified incubator with 5% CO_2_ at 37°C. The medium was exchanged every three days.

H9C2 cells were pretreated with 50 *μ*m salvianolic acid A with 98.15% purity (MedChemExpress, China) for 24 h, as previously described [8]. For establishing an in vitro model of myocardial infarction, H9C2 cells were washed with PBS and maintained in DMEM. Afterwards, they were grown lasting 3 h in an incubator flushed with a gas mixture (1% O_2_, 5% CO_2_ as well as 94% N_2_). Following OGD, they were grown in complete culture medium with 5% CO_2_ and 95% air lasting 6 h. Control cells were grown in DMEM with normoxia.

### 2.8. Cell Viability Assay

Cell viability was assayed with cell counting kit‐8 (CCK‐8) kit (MedChemExpress, China). H9C2 cells were grown in a 96‐well plate (1 × 104 cells/well). 10 *μ*L CCK‐8 reagent was added to each well with H9C2 cells. The plate was cultured at 37°C for 2 h away from light. The optical density was monitored with microplate reader at 450 nm.

### 2.9. Reverse Transcription-Quantitative PCR (RT-qPCR)

Total RNA extraction from H9C2 cells was implemented via TRIzol reagent (Solarbio, China). The RNA content was measured with UV spectrophotometry. Extracted RNA was utilized for reverse transcription and cDNA was synthesized. The qPCR assays were implemented utilizing SYBR Premix Ex Taq II and RT-qPCR detection system. GAPDH was utilized for normalizing mRNA expressions. Relative expressions were computed with 2^−ΔΔCt^. Sequences of primers (SRC, CTNNB1, PIK3CA, AKT1, RELA, EGFR, FYN, ITGB1, MAPK8, and NFKB1) utilized for RT-qPCR are listed in Table 1.

### 2.10. Statistical Analyses

Data were analyzed through appropriate *R* packages and GraphPad Prism 8 software. ANOVA was applied for comparisons between groups, and the difference was statistically significant when *P* < 0.05.

## 3. Results

### 3.1. The Chemical Structure and Potential Targets of Salvianolic Acid A

**Figure 1:**
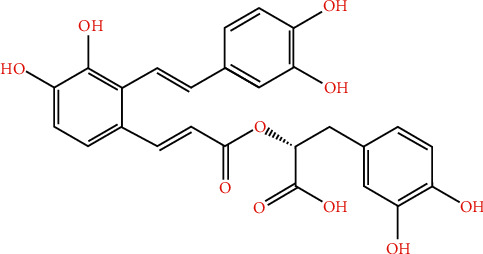
The chemical structure of salvianolic acid A.

**Figure 2:**
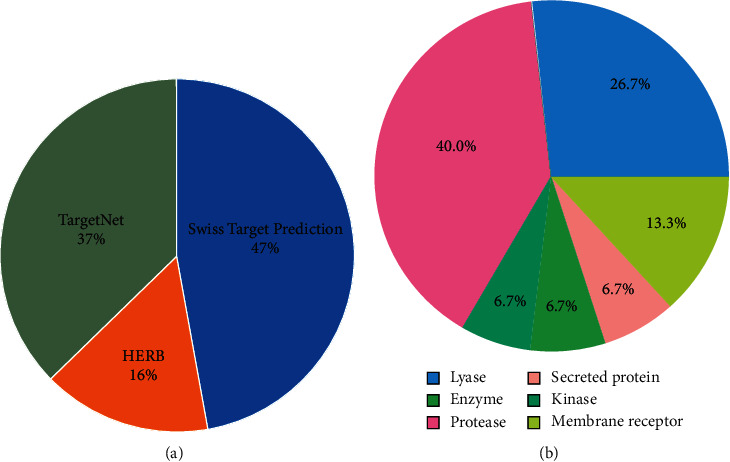
Prediction of potential targets of salvianolic acid A on the basis of SwissTargetPrediction, HERB, and TargetNet web tools. (a) The distribution of SwissTargetPrediction-, HERB-, and TargetNet-predicted targets of salvianolic acid A. (b) Target classes of the top 15 potential targets of salvianolic acid A predicted by the SwissTargetPrediction web tool.

The chemical structure of salvianolic acid A was retrieved from the PubChem database, as illustrated in Figure 1. To determine potential molecular targets of salvianolic acid A, we employed three web tools, comprising SwissTargetPrediction, HERB, and TargetNet databases. As a result, 100 (Table 2), 33 (Table 3), and 79 (Table 4) potential targets of salvianolic acid A were predicted on the basis of SwissTargetPrediction, HERB, and TargetNet databases, respectively. After merging and deduplication, we finally retrieved 180 potential targets of salvianolic acid A (Figure 2(a)). SwissTargetPrediction-, HERB-, and TargetNet-predicted targets of salvianolic acid A occupied 47%, 16%, and 37% of all targets, respectively. Through SwissTargetPrediction web tool, target classes of the top 15 potential targets of salvianolic acid A were analyzed. In Figure 2(b), 40.0% belonged to protease, with 26.7% for lyase, 13.3% for membrane receptor, and with 6.7% for secreted protein, enzyme, or kinase.

### 3.2. Estimation of Targets of Myocardial Infarction

**Figure 3:**
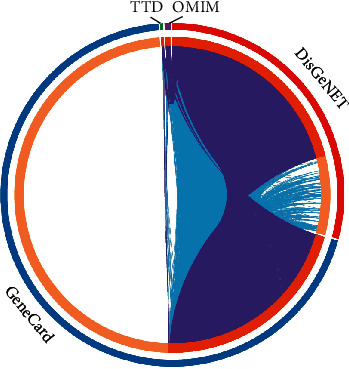
Estimation of targets of myocardial infarction via integrating GeneCards, Online Mendelian Inheritance in Man (OMIM), DisGeNET, and Therapeutic Target Database (TTD) databases.

To estimate the potential targets of myocardial infarction, this study integrated four comprehensive databases comprising GeneCards, OMIM, DisGeNET and TTD. As a result, 4633, 39, 1800, and 36 myocardial infarction-relevant targets were separately retrieved from GeneCards, OMIM, DisGeNET and TTD databases (Figure 3). After merging and deduplication, 5172 disease targets of myocardial infarction were finally obtained.

### 3.3. Identification of Targets Shared by Salvianolic Acid A and Myocardial Infarction

To determine myocardial infarction targets of salvianolic acid A, we took the intersection between targets of salvianolic acid A and myocardial infarction. As illustrated in Figure 4,120 targets shared by salvianolic acid A and myocardial infarction were eventually obtained.

### 3.4. Interactions between Products of Myocardial Infarction Targets of Salvianolic Acid A

**Figure 5:**
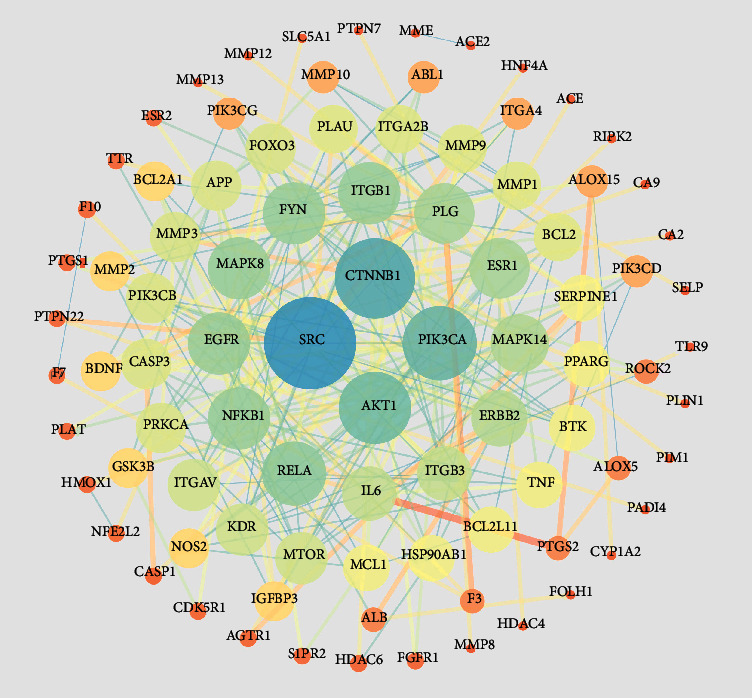
The protein-protein interaction (PPI) network of products of myocardial infarction targets of salvianolic acid A.

**Figure 6:**
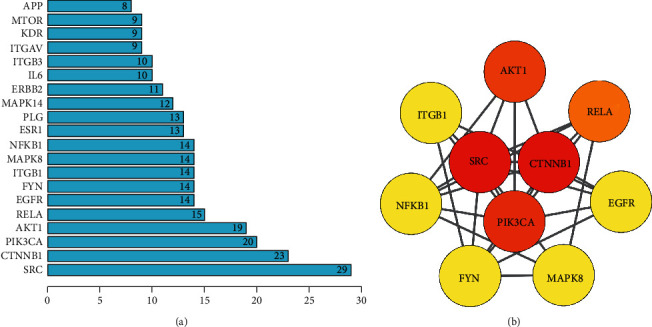
Hub myocardial infarction targets of salvianolic acid A. (a) The top twenty myocardial infarction targets of salvianolic acid A in accordance with the degree. (b) The interaction network of hub myocardial infarction targets of salvianolic acid A.

Through the STRING online tool, we evaluated the interactions between products of myocardial infarction targets of salvianolic acid A.  Figure 5 illustrates their interactions. We computed the degree of each target.  Figure 6(a) visualizes the top twenty myocardial infarction targets of salvianolic acid A in accordance with the degree, comprising SRC, CTNNB1, PIK3CA, AKT1, RELA, EGFR, FYN, ITGB1, MAPK8, NFKB1, ESR1, PLG, MAPK14, ERBB2, IL6, ITGB3, ITGAV, KDR, MTOR, and APP. With cytoHubba plugin, ten hub myocardial infarction targets of salvianolic acid A were determined, covering SRC, CTNNB1, PIK3CA, AKT1, RELA, EGFR, FYN, ITGB1, MAPK8, and NFKB1 (Figure 6(b)).

### 3.5. Biological Functions and Pathways of Myocardial Infarction Targets of Salvianolic Acid A

**Figure 7:**
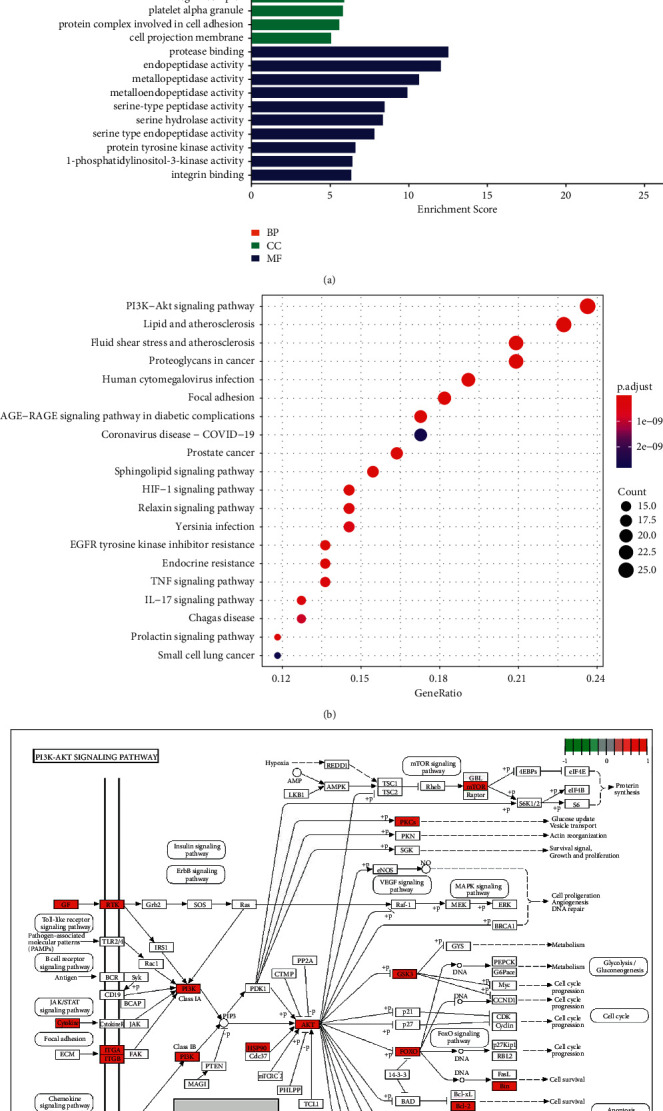
Biological functions and pathways of myocardial infarction targets of salvianolic acid A. (a) The first ten biological processes (BPs), cellular components (CCs), and molecular functions (MFs) of myocardial infarction targets of salvianolic acid A BPs, CCs, and MFs are marked by unique colors. The length of the column is proportional to the enrichment score (b) The first twenty Kyoto Encyclopedia of Genes and Genomes (KEGG) pathways of myocardial infarction targets of salvianolic acid (A) The size of the bubble is proportional to the count of myocardial infarction targets of salvianolic acid A enriched. The closer the color is to red, the smaller the adjusted *p*-value. (c) The map of the PI3K-Akt signaling pathway.

GO enrichment analyses were implemented for probing out biological functions of 120 myocardial infarction targets of salvianolic acid A. In Figure 7(a), the myocardial infarction targets of salvianolic acid A were remarkably linked to biological processes of response to molecule of bacterial origin, response to lipopolysaccharide, cellular response to chemical stress, reactive oxygen species metabolic process, response to oxidative stress, regulation of reactive oxygen species metabolic process, response to reactive oxygen species, peptidyl-serine phosphorylation, cellular response to biotic stimulus, and peptidyl-serine modification. Additionally, cellular components of membrane raft, membrane microdomain, membrane region, vesicle lumen, secretory granule lumen, cytoplasmic vesicle lumen, integrin complex, platelet alpha granule, protein complex involved in cell adhesion, and cell projection membrane were significantly enriched by the myocardial infarction targets of salvianolic acid A. We also found the significant enrichment of molecular functions of protease binding, endopeptidase activity, metallopeptidase activity, metalloendopeptidase activity, serine-type peptidase activity, serine hydrolase activity, serine-type endopeptidase activity, protein tyrosine kinase activity, 1-phosphatidylinositol-3-kinase activity, and integrin binding by the myocardial infarction targets of salvianolic acid A. On the basis of them, the myocardial infarction targets of salvianolic acid A exerted key functions in myocardial infarction.

KEGG pathway enrichment analyses were conducted for unveiling the pathways involved in the myocardial infarction targets of salvianolic acid A. As illustrated in Figure 7(b), myocardial infarction-relevant pathways (PI3K-Akt signaling pathway, lipid and atherosclerosis, fluid shear stress and atherosclerosis, focal adhesion, AGE-RAGE signaling pathway in diabetic complications, sphingolipid signaling pathway, HIF-1 signaling pathway, TNF signaling pathway, IL-17 signaling pathway, etc.) were significantly correlated to the myocardial infarction targets of salvianolic acid A. Especially, the details of PI3K-Akt signaling pathway were visualized, as illustrated in Figure 7(c).

### 3.6. Verification of the Effects and Therapeutic Targets of Salvianolic Acid A in Myocardial Infarction

**Figure 8:**
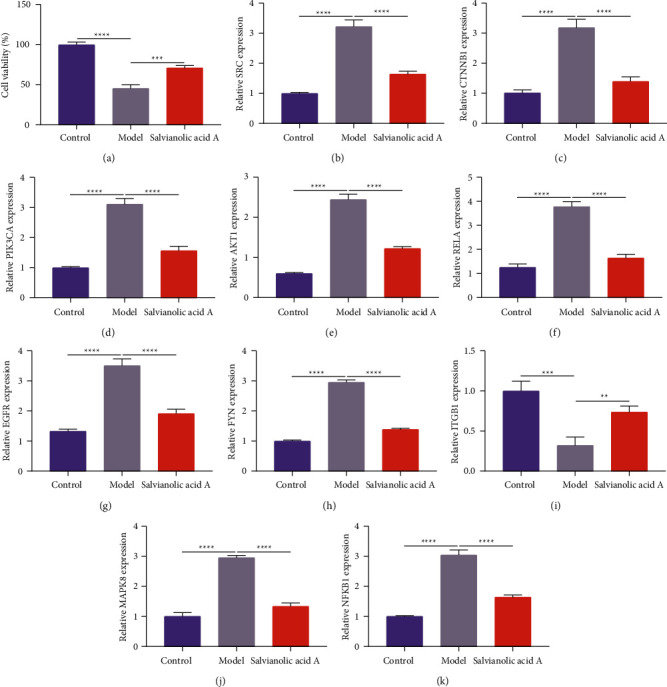
Verification of the effects and therapeutic targets of salvianolic acid A in myocardial infarction. (a) The proliferation of H9C2 cells administrated with normoxia (control), oxygen‐glucose deprivation/reoxygenation (OGD/R) (model), and salvianolic acid A pretreatment. (b–k) Reverse transcription-quantitative PCR of SRC, CTNNB1, PIK3CA, AKT1, RELA, EGFR, FYN, ITGB1, MAPK8, and NFKB1 expressions in H9C2 cells administrated with normoxia (control), OGD/R (model), and salvianolic acid A pretreatment. ^*∗∗*^*p* < 0.01; ^*∗∗∗*^*p* < 0.001;  ^*∗*^ ^*∗*^ ^*∗*^ ^*∗*^*p* < 0.0001.

H9C2 cells were exposed to OGD/R to mimic myocardial infarction. In comparison to normoxia, OGD/R-exposed H9C2 cells presented the reduced proliferation (Figure 8(a)). Pretreatment of 50 *μ*m salvianolic acid A remarkably improved the proliferation of OGD/R-exposed H9C2 cells. Hub myocardial infarction targets of salvianolic acid A were further verified. Compared with normoxia, SRC, CTNNB1, PIK3CA, AKT1, RELA, EGFR, FYN, MAPK8, and NFKB1 expressions were upregulated, and ITGB1 expression was downregulated in OGD/R-exposed H9C2 cells (Figures 8(b)–8(k)). Pretreatment of salvianolic acid A reduced SRC, CTNNB1, PIK3CA, AKT1, RELA, EGFR, FYN, MAPK8, and NFKB1 expressions as well as elevated ITGB1 expression in OGD/R-exposed H9C2 cells.

## 4. Discussion

Salvianolic acid A is extracted from traditional Chinese medicine *Salvia miltiorrhiza*, which is a major water-soluble as well as a biologically active ingredient [28]. The present study employed network pharmacology analyses to uncover the pharmacological targets and therapeutic mechanisms of salvianolic acid A in myocardial infarction. Further, H9C2 cells were administrated with OGD/R to mimic myocardial infarction, and the effects and hub therapeutic targets were confirmed. Thus, our findings unveiled the possible functional mechanisms and pharmacological targets of salvianolic acid A as an antimyocardial infarction therapy.

Through intersecting the targets of salvianolic acid A and myocardial infarction, 120 pharmacological targets of salvianolic acid A in myocardial infarction were determined. Among them, ten hub therapeutic targets were identified, covering SRC, CTNNB1, PIK3CA, AKT1, RELA, EGFR, FYN, ITGB1, MAPK8, and NFKB1. Evidence has demonstrated the functions of the hub therapeutic targets in myocardial infarction. Blockage of SRC can stabilize Flk/cadherin complexing, reduce edema as well as tissue damage after myocardial infarction [29]. Additionally, SRC suppression reverses Cx43 remodeling and improves heart function following myocardial infarction [30]. CTNNB1 protein product *β*-catenin is a key integral part of the canonical Wnt/*β*-catenin pathway. Wnt/*β*-catenin damage response enables to activate the epicardium as well as cardiac fibroblasts for promoting cardiac repair [31]. The blockage of the Wnt/*β*-catenin pathway improves the cardiac function of myocardial infarction [32]. Activated RELA/*p*65 results in myocardial infarction [33], and activation of EGFR-dependent pathway strengthens cardiac fibrosis and exacerbates cardiac dysfunction in myocardial infarction [34]. The basic fibroblast growth factor activates Nrf2-triggered antioxidant defenses through Akt/GSK3*β*/Fyn signaling in myocardial infarction [35]. Endothelial ITGB1 (*β*1 integrin) is essential for the heart to adapt cardiac ischemia and protects from myocardial infarction [36]. ITGB1 upregulation is capable of increasing cardiac function and clinical outcome after myocardial infarction [37, 38]. NFKB1 gene rs28362491 ins/del variation correlates to increased susceptibility to myocardial infarction among Chinese Han patients [39].

Therapeutic targets were significantly linked to myocardial infarction-relevant pathways, such as PI3K-Akt signaling pathway. Evidence demonstrates that salvianolic acid A is regarded as a potential PI3K/Akt inhibitor. For instance, salvianolic acid A enables to attenuate CCl4-triggered liver fibrosis through inactivation of the PI3K-Akt pathway [40]. Moreover, it hinders vasculogenic mimicry formation in human non-small cell lung carcinoma through the PI3K-Akt pathway [41]. Additionally, through suppressing PI3K-Akt pathway, salvianolic acid A triggers cellular apoptosis as well as blocks tumor growth in acute myeloid leukemia [42]. The PI3K-Akt pathway participates in myocardial ischemia/reperfusion damage in diabetic murine models, which is blocked by salvianolic acid A [43]. Salvianolic acid A hinders malignant development of glioma as well as strengthens temozolomide sensitivity through weakening PI3K-Akt pathway [44]. Because the PI3K-Akt pathway plays a key role in administering the process of myocardial infarction, targeting this aberrant signaling pathway as well as improving the pathological manifestation of myocardial infarction remains indispensable [5].

We further verified the effects and therapeutic targets of salvianolic acid A in myocardial infarction in OGD/R-induced H9C2 cells. As expected, salvianolic acid A administration enabled to ameliorate the viability of OGD/R-induced H9C2 cells as well as alter the expression of the hub therapeutic targets. Nevertheless, several limitations should be pointed out. First, the hub therapeutic targets of salvianolic acid A should be verified in myocardial infarction animal models. In our future research, we will further investigate the therapeutic effects as well as pharmacologically relevant targets of salvianolic acid A in myocardial infarction animal models. Second, further clinical trials are urgently needed to verify the therapeutic effects of salvianolic acid A against myocardial infarction.

## 5. Conclusion

The present study unveiled the pharmacological targets and therapeutic mechanisms of salvianolic acid A in myocardial infarction utilizing network pharmacology analyses and in vitro OGD/R H9C2 cellular models of myocardial infarction, which paved the way for further clinical trials.

## Figures and Tables

**Figure 4:**
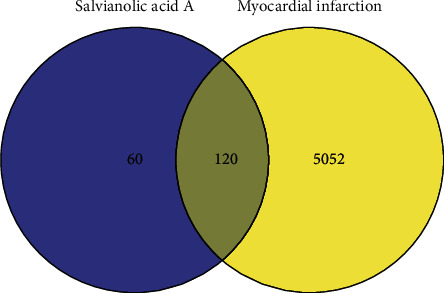
Identification of myocardial infarction targets of salvianolic acid A Venn diagram illustrates the intersection between targets of salvianolic acid A and myocardial infarction.

**Table 1:** Sequences of primers utilized for RT-qPCR.

Target	Primer sequence (5′-3′)
SRC	F : GAGCGGCTCCAGATTGTCAA
	R : CTGGGGATGTAGCCTGTCTGT
CTNNB1	F : AAAGCGGCTGTTAGTCACTGG
	R : CGAGTCATTGCATACTGTCCAT
PIK3CA	F : CCACGACCATCATCAGGTGAA
	R : CCTCACGGAGGCATTCTAAAGT
AKT1	F : AGCGACGTGGCTATTGTGAAG
	R : GCCATCATTCTTGAGGAGGAAGT
RELA	F : ATGTGGAGATCATTGAGCAGC
	R : CCTGGTCCTGTGTAGCCATT
EGFR	F : AGGCACGAGTAACAAGCTCAC
	R : ATGAGGACATAACCAGCCACC
FYN	F : ATGGGCTGTGTGCAATGTAAG
	R : GAAGCTGGGGTAGTGCTGAG
ITGB1	F : CCTACTTCTGCACGATGTGATG
	R : CCTTTGCTACGGTTGGTTACATT
MAPK8	F : TGTGTGGAATCAAGCACCTTC
	R : AGGCGTCATCATAAAACTCGTTC
NFKB1	F : AACAGAGAGGATTTCGTTTCCG
	R : TTTGACCTGAGGGTAAGACTTCT
GAPDH	F : CTGGGCTACACTGAGCACC
	R : AAGTGGTCGTTGAGGGCAATG

**Table 2:** Potential molecular targets of salvianolic acid A by the SwissTargetPrediction web tool.

Target	Target Class	Target	Target Class
CA12	Lyase	AURKA	Kinase
CA4	Lyase	AKR1B10	Enzyme
CA7	Lyase	DHFR	Oxidoreductase
CA2	Lyase	F3 F7	Protease
MMP1	Protease	CASP1	Protease
AKR1B1	Enzyme	AGTR1	Family A *G* protein-coupled receptor
FYN	Kinase	HMGCR	Oxidoreductase
TTR	Secreted protein	THRB	Nuclear receptor
MMP9	Protease	ITGB5 ITGAV	Membrane receptor
MMP12	Protease	IMPDH1	Oxidoreductase
MME	Protease	ERBB2	Kinase
SLC28A2	Electrochemical transporter	LCK	Kinase
ADAMTS4	Protease	HSP90AB1	Other cytosolic protein
ADORA3	Family A *G* protein-coupled receptor	LDHA	Enzyme
ACE	Protease	THRA	Nuclear receptor
SLC5A1	Electrochemical transporter	MMP8	Protease
GRM2	Family C *G* protein-coupled receptor	ECE1	Protease
ITGB7 ITGA4	Membrane receptor	PTGDR2	Family A *G* protein-coupled receptor
F7	Protease	CA14	Lyase
ALB	Secreted protein	DHODH	Oxidoreductase
MMP2	Protease	TDP1	Enzyme
PIM1	Kinase	GALK1	Enzyme
SELP	Adhesion	ABL1	Kinase
MMP13	Protease	PADI1	Enzyme
SELL	Adhesion	MAP3K9	Kinase
IGFBP3	Secreted protein	F3	Surface antigen
KDM4C	Eraser	MMP10	Protease
SLC5A2	Electrochemical transporter	PADI4	Enzyme
MMP3	Protease	AMPD3	Enzyme
SELE	Adhesion	ITGA2B ITGB3	Membrane receptor
ITGB1 ITGA4	Membrane receptor	C3AR1	Family A *G* protein-coupled receptor
HCAR2	Family A *G* protein-coupled receptor	TYR	Oxidoreductase
CA1	Lyase	ADK	Enzyme
TYMS	Transferase	AMPD2	Enzyme
SLC6A2	Electrochemical transporter	EGFR	Kinase
AKR1C2	Enzyme	YARS	Enzyme
APP	Membrane receptor	ITGAV ITGB3	Membrane receptor
ITGAV ITGB1	Membrane receptor	SLC5A4	Electrochemical transporter
MAPK8	Kinase	SRD5A1	Oxidoreductase
ROCK2	Kinase	PADI2	Enzyme
GART	Ligase	PADI3	Enzyme
PRKCA	Kinase	EPHA2	Kinase
CASP3	Protease	FGFR1	Kinase
F10	Protease	LDHB	Enzyme
CA9	Lyase	BTK	Kinase
SRC	Kinase	SLC28A3	Electrochemical transporter
KDR	Kinase	CA5A	Lyase
SLC29A1	Electrochemical transporter	CA6	Lyase
ESR1	Nuclear receptor	CA5B	Lyase
ACE2	Protease	CA13	Lyase

**Table 3:** Potential molecular targets of salvianolic acid A by the HERB database.

Paper ID	Target ID	Target name	PubMed ID
HBREF001977	HBTAR005574	BCL2L11	31193821
HBREF001977	HBTAR001405	FOXO3	31193821
HBREF001977	HBTAR003003	PIK3CA	31193821
HBREF001977	HBTAR003004	PIK3CB	31193821
HBREF001977	HBTAR003006	PIK3CD	31193821
HBREF001977	HBTAR003007	PIK3CG	31193821
HBREF001977	HBTAR000130	AKT1	31193821
HBREF001978	HBTAR003991	TAGLN	30361065
HBREF001979	HBTAR002705	NFKB1	28303221
HBREF001980	HBTAR000636	CDK5	27609227
HBREF001980	HBTAR001009	DCX	27609227
HBREF001980	HBTAR002705	NFKB1	27609227
HBREF001980	HBTAR003003	PIK3CA	27609227
HBREF001980	HBTAR003004	PIK3CB	27609227
HBREF001980	HBTAR003006	PIK3CD	27609227
HBREF001980	HBTAR003007	PIK3CG	27609227
HBREF001980	HBTAR000374	BDNF	27609227
HBREF001980	HBTAR000360	BCL2	27609227
HBREF001980	HBTAR004910	CDK5R1	27609227
HBREF001980	HBTAR000920	CTNNB1	27609227
HBREF001980	HBTAR001710	GSK3B	27609227
HBREF001981	HBTAR000130	AKT1	24486344
HBREF001981	HBTAR001437	MTOR	24486344
HBREF001981	HBTAR001851	HMOX1	24486344
HBREF001981	HBTAR002700	NFE2L2	24486344
HBREF001982	HBTAR000874	MAPK14	24033467
HBREF001982	HBTAR000151	ALOX5	24033467
HBREF001982	HBTAR001851	HMOX1	24033467
HBREF001982	HBTAR003295	PTGS2	24033467
HBREF001982	HBTAR002055	IL6	24033467
HBREF001982	HBTAR002705	NFKB1	24033467
HBREF001982	HBTAR002737	NOS2	24033467
HBREF001982	HBTAR004140	TNF	24033467

**Table 4:** Potential molecular targets of salvianolic acid A by TargetNet web service.

Uniprot ID	Protein
P51661	Corticosteroid 11-beta-dehydrogenase isozyme 2
P10826	Retinoic acid receptor beta
Q86TI2	Dipeptidyl peptidase 9
Q923Y8	Trace amine-associated receptor 1
P35463	Endothelin B receptor
P13497	Bone morphogenetic protein 1
P20444	Protein kinase C alpha type
P23978	Sodium- and chloride-dependent GABA transporter 1
P10276	Retinoic acid receptor alpha
P19634	Sodium/hydrogen exchanger 1
P06737	Glycogen phosphorylase, liver form
P04035	3-Hydroxy-3-methylglutaryl-coenzyme A reductase
O14939	Phospholipase D2
P56658	Adenosine deaminase
P19320	Vascular cell adhesion protein 1
P48039	Melatonin receptor type 1A
P08473	Neprilysin
Q29463	Anionic trypsin
P05106	Integrin beta-3
Q920D2	Dihydrofolate reductase
Q07422	Bifunctional dihydrofolate reductase-thymidylate synthase
P05364	Beta-lactamase
O00763	Acetyl-CoA carboxylase 2
Q04609	Glutamate carboxypeptidase 2
P16257	Translocator protein
P28702	Retinoic acid receptor RXR-beta
P00375	Dihydrofolate reductase
P26684	Endothelin-1 receptor
Q9QYJ6	cAMP and cAMP-inhibited cGMP 3′,5′-cyclic phosphodiesterase 10A
Q9UHL4	Dipeptidyl peptidase 2
P20292	Arachidonate 5-lipoxygenase-activating protein
P62943	Peptidyl-prolyl cis-trans isomerase FKBP1A
P19156	Potassium-transporting ATPase alpha chain 1
P50579	Methionine aminopeptidase 2
P13631	Retinoic acid receptor gamma
Q8WW43	Gamma-secretase subunit APH-1B
P62942	Peptidyl-prolyl cis-trans isomerase FKBP1A
Q8TDS4	Hydroxycarboxylic acid receptor 2
P51639	3-Hydroxy-3-methylglutaryl-coenzyme A reductase
P48443	Retinoic acid receptor RXR-gamma
O14842	Free fatty acid receptor 1
Q92542	Nicastrin
P41231	P2Y purinoceptor 2
P31639	Sodium/glucose cotransporter 2
P00491	Purine nucleoside phosphorylase
P55157	Microsomal triglyceride transfer protein large subunit
Q9NZ42	Gamma-secretase subunit PEN-2
Q01727	Melanocyte-stimulating hormone receptor
P47820	Angiotensin-converting enzyme
Q63470	Dual specificity tyrosine-phosphorylation-regulated kinase 1A
P16753	Capsid scaffolding protein
Q02769	Squalene synthase
P49768	Presenilin-1
P19099	Cytochrome P450 11B2, mitochondrial
Q96BI3	Gamma-secretase subunit APH-1A
Q8TDV5	Glucose-dependent insulinotropic receptor
O77636	Disintegrin and metalloproteinase domain-containing protein 17
P40238	Thrombopoietin receptor
P13945	Beta-3 adrenergic receptor
P49430	Thromboxane-A synthase
Q7TMR0	Lysosomal Pro-X carboxypeptidase
P16184	Dihydrofolate reductase
O95822	Malonyl-CoA decarboxylase, mitochondrial
Q05469	Hormone-sensitive lipase
P34976	Type-1 angiotensin II receptor
P05093	Steroid 17-alpha-hydroxylase/17,20 lyase
O70536	Sterol O-acyltransferase 1
Q95323	Carbonic anhydrase 4
P49682	C-X-C chemokine receptor type 3
P14740	Dipeptidyl peptidase 4
Q16602	Calcitonin gene-related peptide type 1 receptor
P55263	Adenosine kinase
P31648	Sodium- and chloride-dependent GABA transporter 1
P09958	Furin
P08842	Steryl-sulfatase
P15538	Cytochrome P450 11B1, mitochondrial
P30557	Prostaglandin E2 receptor EP3 subtype
P13516	Acyl-CoA desaturase 1
Q62053	Prostaglandin E2 receptor EP2 subtype

## Data Availability

The datasets analyzed during the current study are available from the corresponding author on reasonable request.

## Abbreviations:

OGD/R: Oxygen‐glucose deprivation/reoxygenation
OMIM: Online Mendelian Inheritance in Man
TTD: Therapeutic Target Database
PPI: Protein-protein interaction
GO: Gene ontology
BPs: Biological processes
CCs: Cellular components
MFs: Molecular functions
KEGG: Kyoto Encyclopedia of Genes and Genomes
CCK‐8: Cell counting kit‐8
RT-qPCR: Reverse transcription-quantitative PCR.
